# Multifunctional, Multivalent PIC Polymer Scaffolds for Targeting Antigen-Specific, Autoreactive B Cells

**DOI:** 10.1021/acsbiomaterials.1c01395

**Published:** 2022-03-08

**Authors:** Hendy Kristyanto, Miles D. Holborough-Kerkvliet, Lianne Lelieveldt, Yvonne Bartels, Roel Hammink, Karin A. J. van Schie, Rene E. M. Toes, Kimberly M. Bonger, Hans Ulrich Scherer

**Affiliations:** † Department of Rheumatology, Leiden University Medical Center, Albinusdreef 2, 2333 ZA Leiden, The Netherlands; ‡ Department of Synthetic Organic Chemistry, Radboud University, Heyendaalseweg 135, 6525 AJ Nijmegen, The Netherlands; § Department of Tumor Immunology, Radboud Institute for Molecular Life Sciences, Division of Immunotherapy, Radboud University Medical Center, 6525 GA Nijmegen, Netherlands; ∥ Oncode Institute, Radboud University Medical Center, 6525 GA Nijmegen, Netherlands

**Keywords:** rheumatoid arthritis, anti-citrullinated protein antibodies, polyisocyanopeptides, cyclic-citrullinated peptide, CD22

## Abstract

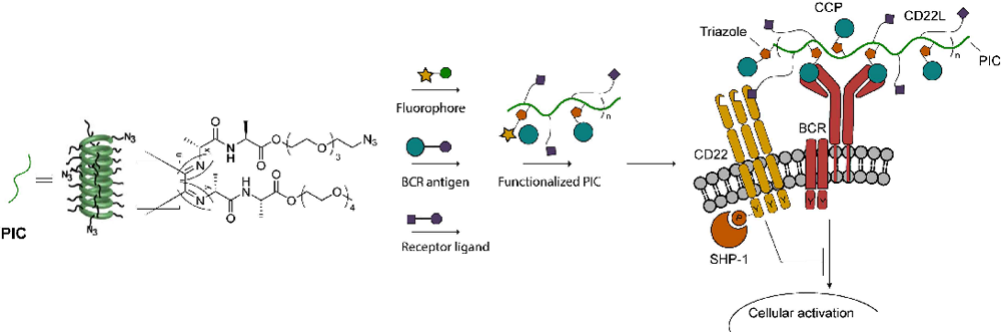

Multivalent scaffolds that carry multiple molecules with immunophenotyping or immunomodulatory properties are invaluable tools for studying and modulating specific functions of human immune responses. So far, streptavidin–biotin-based tetramers have been widely used for B-cell immunophenotyping purposes. However, the utility of these tetramers is limited by their tetravalency, the inherent immunogenicity of streptavidin (a bacterial protein that can potentially be recognized by B cells), and the limited feasibility to functionalize these reagents. This has rendered tetramers suboptimal for studying rare, in particular, antigen-specific B-cell populations in the context of clinical applications. Here, we used polyisocyanopeptides (PICs), multivalent polymeric scaffolds functionalized with around 50 peptide antigens, to detect autoreactive B cells in the peripheral blood of patients with rheumatoid arthritis. To explore the potential immunomodulatory functionalities, we functionalized PICs with autoantigenic peptides and a trisaccharide CD22 ligand to inhibit autoreactive B-cell activation through interference with the B-cell receptor activation pathway, as evidenced by reduced phospho-Syk expression upon PIC binding. Given the possibilities to functionalize PICs, our data demonstrate that the modular and versatile character of PIC scaffolds makes them promising candidates for future clinical applications in B-cell-mediated diseases.

## Introduction

Aberrant B cells that recognize and attack the body’s own tissues are drivers of pathology in many autoimmune diseases, including rheumatoid arthritis (RA), as evidenced by the efficacy of B-cell targeting therapies. If left untreated, RA is characterized by progressive synovial inflammation, cartilage and bone destruction, and functional disability. Anti-citrullinated protein antibody (ACPA) responses are a hallmark of RA and target (self-)proteins, in which arginine residues have been converted to citrulline by post-translational modification. ACPA can be detected in serum years before the onset of clinically detectable arthritis. Their presence prognosticates disease onset and the development of severe joint erosions in established RA.^1^

Previously, we showed that ACPA-expressing memory B cells (MBCs) display an activated and proliferative phenotype at the onset of RA that persists throughout the course of chronic disease despite successful suppression of local and systemic inflammation by disease-modifying anti-rheumatic drugs.^2^ These autoreactive MBCs likely exert pathogenic effector functions through the co-stimulation of T cells, the production of pro-inflammatory cytokines, and the recruitment of neutrophils to sites of the local inflammation.^1^ The involvement of these cells in the inflammatory disease process is additionally supported by the therapeutic efficacy of rituximab, a broadly B-cell depleting agent approved for clinical use. Rituximab depletes the naïve and MBC compartments but leaves the compartment of antibody-secreting cells largely intact.^3,4^ Till date, the triggers initiating the generation of ACPA-expressing B cells and the factors/antigens that maintain their chronic activation remain largely unclear. Additionally, ways to specifically target ACPA-expressing MBCs, which would alleviate the risks associated with broadly immunosuppressive interventions such as rituximab, are missing.

**Figure 1:**
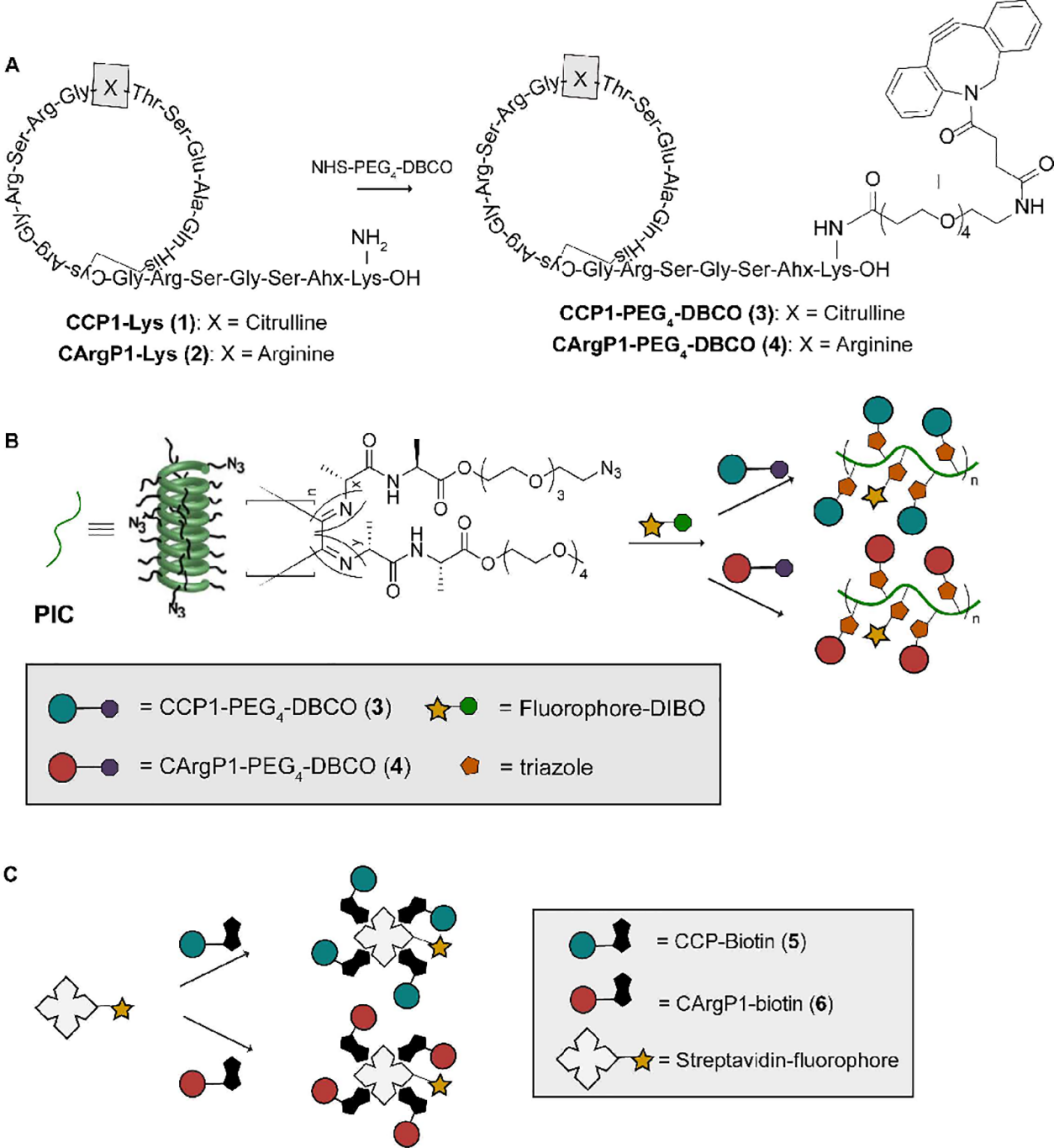
Overview of multivalent antigen-carrying PICs (B) and tetrameric antigen carrying streptavidin SA (C). (A) Structure of CXP [X = citrulline (CCP) or arginine (CArgP)]. CXP was synthesized with a lysine at the C-terminus, which can be modified using an NHS-PEG4-DBCO for cycloaddition purposes to the PIC polymers. (B) Schematic overview of the synthesis approach of CXP-conjugated PICs. All PICs were first reacted with DBCO-biotin and subsequently with DIBO fluorophores and with one of the respective antigen peptides to form the multivalent antigen structure. (C) Overview of the previously published tetrameric antigen SA approach.

Here, we used ACPA-expressing B cells as a surrogate for antigen-specific, autoreactive B-cell responses in human autoimmune diseases and set out to improve the detectability of autoreactive B cells in patients while simultaneously developing tools to specifically target these cells. Major challenges that have so far hampered the study of human autoreactive B cells relate to their very low frequency in peripheral blood, as well as the variable affinity of the autoreactive B-cell receptors (BCRs) for their cognate autoantigens.^5^ We previously developed a streptavidin (SA)-biotin-based tetramer approach to detect ACPA-expressing B cells in peripheral blood and synovial fluid.^2^ We now employed polyisocyanopeptides (PICs), which are multivalent polymeric scaffolds that can be functionalized with peptides, fluorophores, and additional molecules capable of modulating B-cell function. PICs are synthetic, water-soluble polymers that form stable helical filaments through internal hydrogen bonding along the polymer backbone facilitated by the peptide bonds in the side chains (Figure 1).^6^ PICs are semiflexible, have a length of several hundred nanometers,^7^ and may be non-immunogenic in mouse models, making them suitable for in vivo use.^8−10^ Moreover, data suggest that the semiflexible nature of PICs is advantageous for the interaction with cell surface receptors, which frequently need to cluster for the downstream (mechano-)transduction of cell signaling.^11,12^ Addition of azide monomers in the synthesis of PICs results in a readily modifiable polymer that can be functionalized with more than 100 copies of molecules with immunophenotyping or immunotherapeutic properties. Multivalent, anti-CD3 antibody-conjugated PICs, for example, resulted in greater and prolonged activation of T cells compared to the effect induced by a single unconjugated anti-CD3-antibody.^13^ In addition, combining two immunostimulatory molecules, anti-CD3 and anti-CD28 antibodies, on one PIC molecule was found to be superior to a combination of monofunctional anti-CD3 PIC and anti-CD28 PIC for T-cell activation.^11^ These data demonstrate the importance of both multivalency and the nano-scale spatial arrangement of immunostimulatory molecules to exert their combined immunotherapeutic properties.

To test and exploit these findings and the technical possibilities offered by PICs in the context of (auto)reactive B cells, we functionalized PICs with an autoreactive cyclic citrullinated peptide (CCP) antigen, a fluorophore, and a trisaccharide ligand (CD22L) for the immunomodulatory receptor molecule CD22 expressed by B cells. We show that PICs can be used as versatile scaffolds to identify ACPA-expressing B cells and demonstrate that the polymers can be used to specifically inhibit B-cell function by co-engaging the BCR and CD22. These data highlight the versatility and applicability of PICs to study and target autoreactive B cells, extending beyond the current possibilities offered by streptavidin-biotin-based tetramer approaches.

## Results and Discussion

### Synthesis of PICs Containing Citrullinated Peptides and Fluorophores

PICs are based on water-soluble polyisocyanopeptide co-polymers carrying non-functional methoxy and functional azide groups. All PICs were synthesized in line with previously published methods.^11,12^ In short, methoxy and azide isocyanopeptide monomers were polymerized using a nickel catalyst, which resulted in azide-functionalized PICs with an average length of ∼400 nm (Figure 1). The methoxy/azide ratio was determined to be 30:1, statistically yielding functional azide groups every 3.5 nm. Roughly half of the azides were used in a strain-promoted azide–alkyne cycloaddition with DBCO–PEG_4_–biotin.^14^ The biotinylation of PICs is used for purification purposes or to secondary stain the PICs with streptavidin in fluoresence-activated cell sorting (FACS) analysis.^7^

To study the application of PICs for the detection of ACPA-expressing B cells, we prepared a set of fluorescently labeled PICs containing CCP and arginine-containing control peptides (CArgP, Figure 1B), as well as tetrameric CCP-SA and CArgP-SA containing the same fluorophores (Figure 1C).^15^ CCP1, a first-generation CCP analogue (termed CCP hereafter), is a known antigen of ACPA^16^ and is recognized by both patient-derived ACPA and immortalized ACPA-expressing B cells.^17,18^ In CArgP, the citrulline residue that is essential for recognition by ACPA is replaced by an arginine. Besides the PIC constructs, we prepared fluorescent streptavidin (SA) conjugates loaded with biotin-functionalized CCP or CArgP to compare our results with the current benchmark (Figure 1C).

CCP and CArgP peptides were prepared by solid phase peptide synthesis and equipped with a C-terminal lysine residue for further functionalization. The peptides were cleaved from the resin, cyclized via their N-terminus, purified, and reacted to NHS-PEG_4_-DBCO (Figure 1A). Prior to antigen functionalization, the PICs were equipped with a DIBO-Alexa Fluor 647 (AF647). The remaining azides were functionalized with either DBCO-CCP (**3**) or DBCO-CArgP (**4**, Figure 1B). Biotinylated CCP (**5**) and CArgP (**6**) were conjugated to AF647-streptavidin (SA-AF647) and used as comparators.^17,18^

We first evaluated the binding properties and determined the optimal peptide/fluorophore ratio of the modified PIC conjugates in a serial dilution experiment measured by flow cytometry (Figure S1). For this, we estimated the *K*_D_ values for the binding of CCP-PIC-AF647 (10:1 and 2:1) and CCP-SA-AF647 to HEK cells expressing an ACPA B-cell receptor (HEK^ACPA-TM^) and cells not expressing any B-cell receptor (HEK^WT^, Figure S2A-B). Based on these data, CCP-AF647-PICs with a CCP/AF647 ratio of 2:1 showed the highest binding affinity to HEK^ACPA-TM^ cells with low background staining of HEK^WT^ cells and were used for subsequent experiments with ACPA-expressing primary B cells. Notably, both PIC conjugates showed lower *K*_D_ values for HEK^ACPA-TM^ cells while less background labeling to HEK^WT^ cells, indicating a better signal-to-noise ratio compared to the SA conjugates (Figure S2A-B).

### CCP-Modified PICs and SA Specifically Bind to ACPA-Expressing B Cells

**Figure 2:**
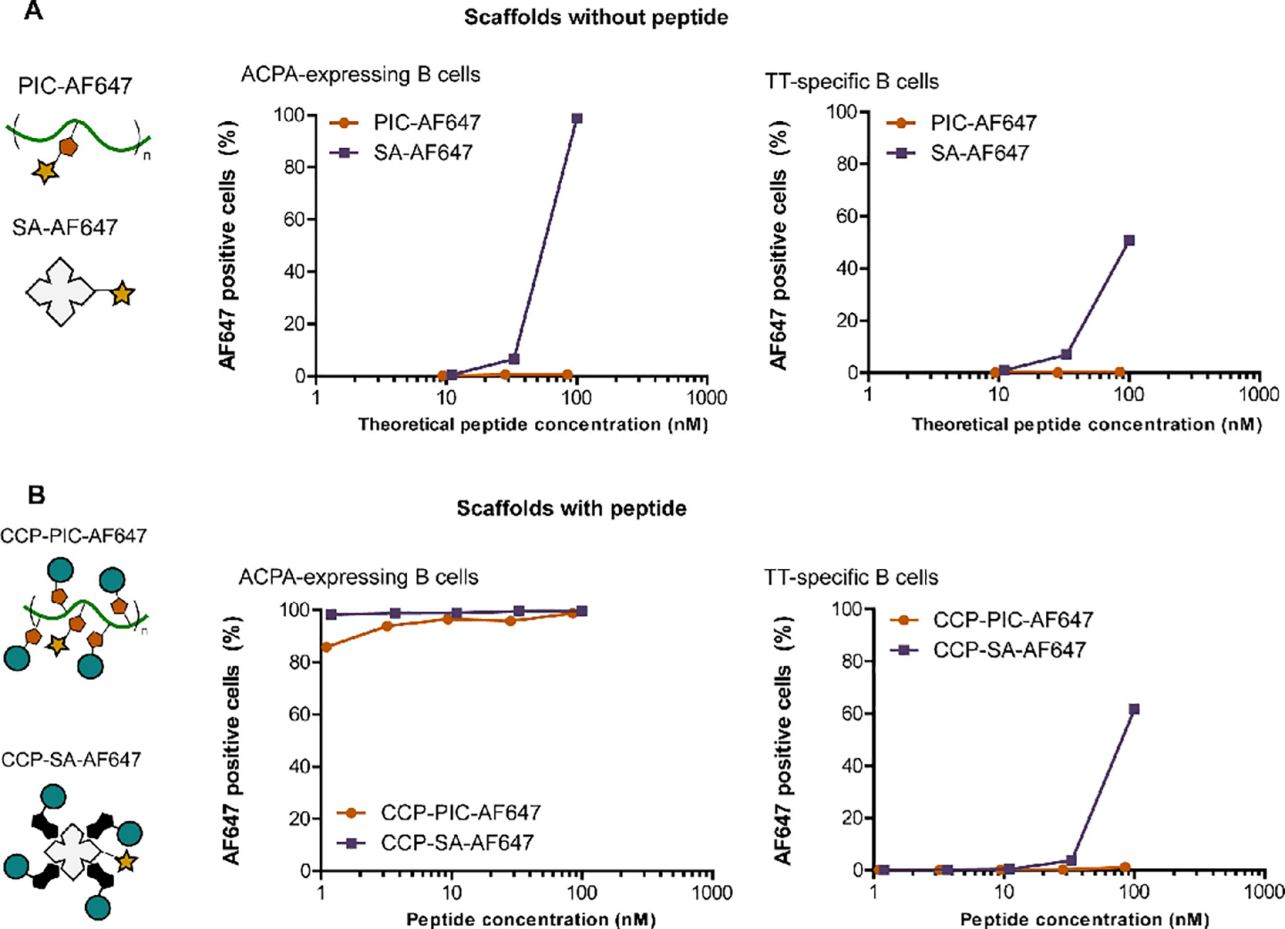
Flow cytometry experiments performed on immortalized primary ACPA-expressing B cells. PIC and SA concentrations were corrected for the number of peptides carried on the respective scaffolds (schematic representation shown). (A) Percentage of ACPA-expressing and TT-specific B cells stained with increasing concentrations of PIC-AF647 and SA-AF647. Theoretical peptide concentration in 2A refers to the theoretical peptide concentration that the PIC backbone would have carried, if functionalized with CCP. (B) Percentage of ACPA-expressing and TT-specific B cells stained with increasing concentrations of CCP-PIC-AF647 and CCP-SA-AF647.

Next, we evaluated the binding and the level of background staining of our PIC conjugates to B cells. For this, we used ACPA-expressing, GFP-positive B cells obtained from one RA-patient that were immortalized in vitro.^19^ As a negative control, we used GFP-positive immortalized B cells expressing a tetanus toxoid (TT)-specific B-cell receptor that is not reactive toward citrullinated CCP antigens.^2^ PIC-AF647 and SA-AF647 were synthesized with and without peptides to evaluate non-specific binding to B cells (Figure 2A,B respectively, Figure S3A). For conditions without peptides, similar PIC-AF647 and SA-AF647 concentrations were used as the CCP-functionalized counterparts (termed “theoretical peptide concentration,” Figure 2A). PICs functionalized with peptide/fluorophore ratios of 10:1 were used in background staining experiments. Due to the 10:1 peptide/fluorophore ratio used for PIC labeling, the PICs used can carry five times more peptides than SA per fluorophore molecule, resulting in a fivefold lower fluorophore concentration for PIC-AF647 scaffolds than that for SA-AF647 (calculations are given in Supporting Information). ACPA-expressing and TT-specific B cells were incubated with unmodified PIC-AF647 and SA-AF647 in a serial dilution experiment starting at the (theoretical) peptide concentration of 100 nM. On both ACPA-expressing and TT-specific B cells, SA-AF647 showed a concentration-dependent background signal that was not observed for any of the PIC-AF647 concentrations. The discrepancy in the background signal between PIC-AF647 and SA-AF647 could not be explained by the fact that the SA-AF647 conditions contained five times higher concentrations of AF647, resulting from equalizing the peptide concentrations, as the SA-AF647 background exceeded that of the PICs by more than fivefold. This suggests that the non-specific binding is caused by SA and not by AF647. Moreover, due to the multivalent nature of PICs, lower concentrations of PICs are required in experiments to achieve the same concentration of peptide compared to tetramers.

Next, we compared the binding of CCP-PIC-AF647 and CCP-SA-AF647 carrying the ACPA-reactive CCP antigen to ACPA-expressing B cells (Figures 2B, S3B). At all concentrations tested, the CCP-SA-AF647 and CCP-PIC-AF647 stained almost all ACPA-expressing B cells. For the TT-specific B cells, we observed a background signal for CCP-SA-AF647 at higher concentrations, in line with the binding signal observed using SA-AF647 lacking CCP. Together, we conclude that both CCP-PIC-AF647 and CCP-SA-AF647 can be used to stain ACPA-expressing B cells. Likewise, despite the higher antigen-valency of PICs, both antigen-expressing tetramers and PICs stained ACPA-expressing B cells equally well, but the SA tetramers showed more non-specific binding at higher concentrations.

### CCP-Modified PICs and SA Fully Detect ACPA-Expressing B Cells in PBMCs

The identification of rare, antigen-specific B-cell populations by flow cytometry with reliable separation of fluorescent signals from background using SA-tetramer antigens requires a double staining approach with differentially labelled antigen multimers.^18^ To evaluate this approach using PICs, we generated additional PICs carrying CCP and AF594 (CCP-PIC-AF594) with similar peptide-to-fluorophore ratios (2:1) to the previously prepared PICs carrying AF647. In addition, a negative control PIC scaffold functionalized with CArgP and AF405 at a ratio of 2:1 was synthesized to ensure specificity of the staining signal for the citrullinated peptide variant. These PICs were synthesized similar to previous PICs.

**Figure 3:**
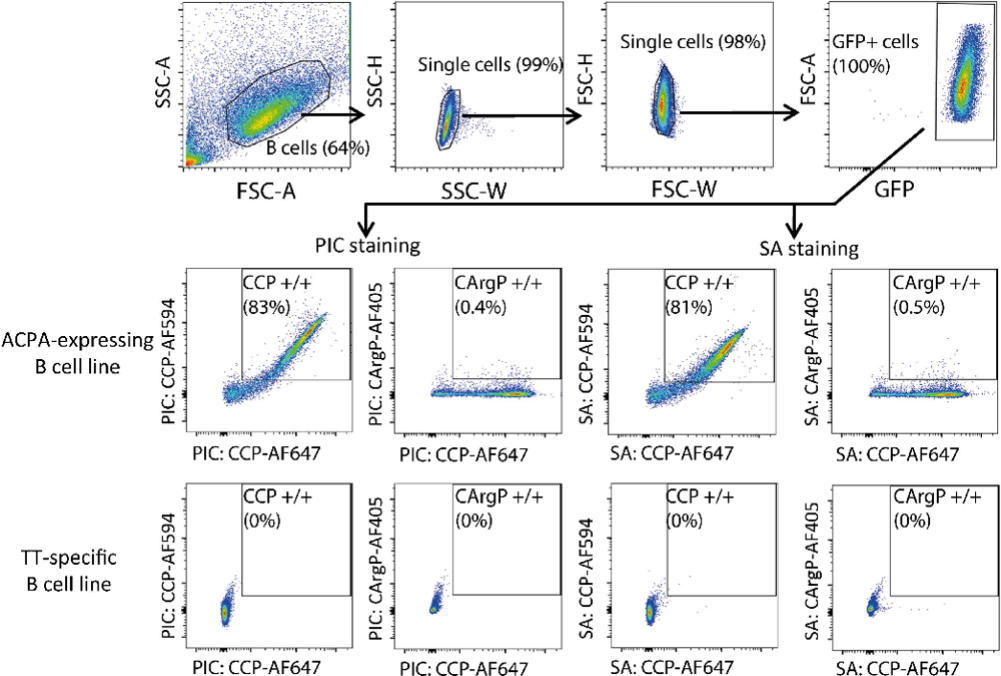
Detection of immortalized ACPA-expressing B cells using a combination of fluorescently labeled CCP- or CArgP-PICs. GFP-positive, immortalized ACPA-expressing, and TT-specific B cells were stained with a combination of fluorophore-labeled PIC containing CCP and its arginine variant control (CArgP) or a combination of fluorophore-labeled CCP- and CArgP streptavidin tetramer (SA).

In order to detect both ACPA-expressing and TT-specific B cells, the differentially labeled PICs, CCP-AF647-PIC, CCP-AF594-PIC, and control CArgP-AF405-PIC were used at a concentration of 85 nM to stain both ACPA-expressing and TT-specific B cells (Figure 3). This concentration showed the best signal-to-noise ratio in prior experiments (Figure S1). For comparison of the previously validated staining method of B cells using SA, a combination of CCP-AF647-SA, CCP-AF594-SA, and control CArgP-AF405-SA was used to stain the same cell lines, serving as a reference for the previously validated staining method for the identification of ACPA-expressing B cells.^17^ The combination of PICs clearly detected the ACPA-expressing B-cell clone. Importantly, TT-specific negative control B cells were not stained, showing the specificity of this approach (Figure 3). These findings were similar to the staining pattern observed using conventional SA tetramers, confirming that the PICs in different fluorochrome combinations can be used to reliably identify ACPA-expressing B cells.

**Figure 4:**
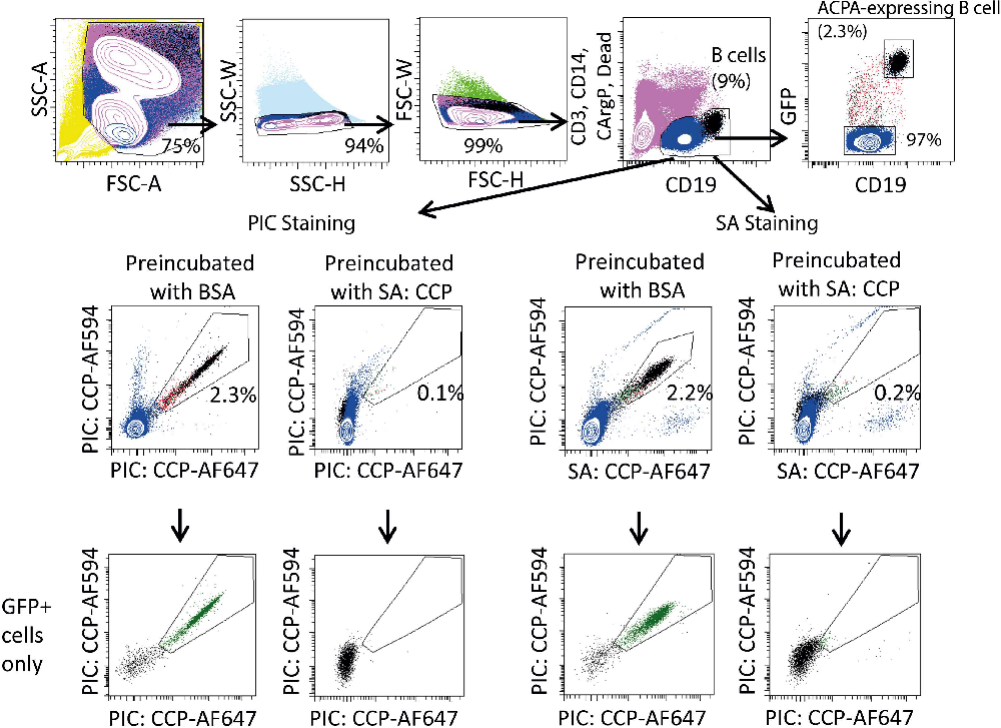
Comparison of fluorescently labeled CCP multivalent scaffolds based on PIC and streptavidin (SA) in the identification and characterization of rare ACPA-expressing B cells. Both PIC and SA-based fluorescently labeled CCP scaffolds can specifically identify 10,000 immortalized ACPA-expressing B cells mixed in with 10 million PBMCs from a healthy donor. Pre-treatment of the cell mix with concentrated unlabeled CCP-SA eliminated most of CCP double positive population.

Inspired by the data above, we next wished to determine whether PICs can, indeed, be used to detect ACPA-expressing B cells in a clinical setting, a setting in which the frequency of antigen-specific B cells is very low (i.e., 1 in 2500–10,000 total B cells^2^). Therefore, 10,000 immortalized ACPA-expressing B cells were mixed in with 10 million PBMCs from healthy donors. Importantly, the immortalized B cells also expressed GFP, allowing their detection independently of the PIC conjugates. As depicted in Figure 4, the combination of 85 nM differentially labeled PIC conjugates readily detected immortalized GFP-positive, ACPA-expressing B cells in the mixture of PBMCs. 2.3% of all B cells was identified to be ACPA-expressing B cells in this setting, translating to approximately 0.1% out of all PBMCs, as expected. Importantly, the vast majority GFP+ cells interacted with the antigen-expressing PICs. Blocking with an excess of unlabeled CCP-SA was used to confirm the specificity of binding (Figure 4). Similar percentages of ACPA-expressing B cells were observed in samples stained with the combined CCP-SA and CCP-PICs and show the high percentage of recovery of rare B cells in a sample of PBMCs using PICs and SA.

### PICs Identify Equal Numbers of Patient ACPA-Expressing B Cells and can be Used for Immunophenotyping of B-Cell Populations

**Figure 5:**
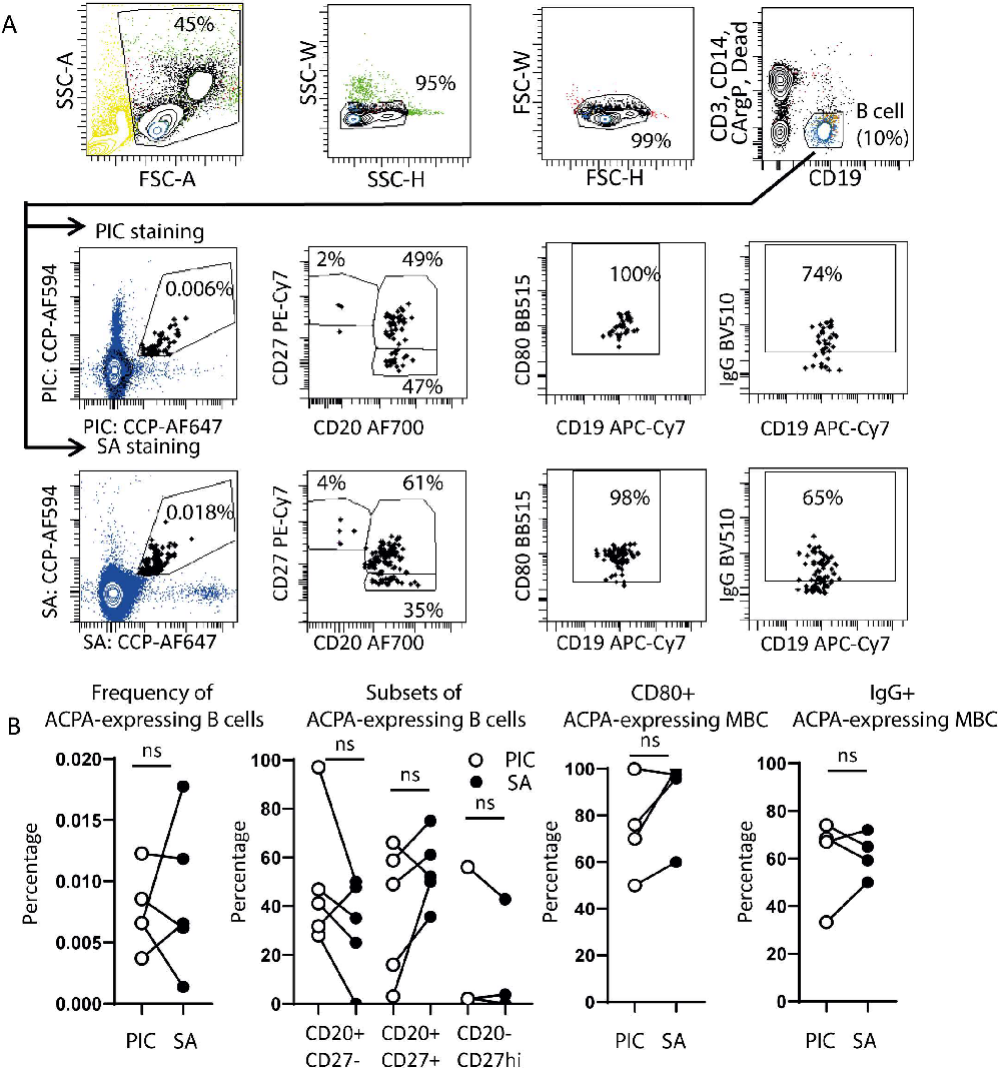
ACPA-expressing B cells identified with both CCP-PIC-AF647 and CCP-SA-AF647 lead to comparable frequencies of cells (*n* = 5), subset characteristics (CD20 and CD27, *n* = 5), activation marker expression (CD80, *n* = 4), and B-cell receptor isotype usage (IgG, *n* = 4) in RA patient’s peripheral blood. Each dot represents one patient sample. Connected dots depict data from individual patient samples. Statistical differences between groups were determined using the Wilcoxon test. (ns, *p* > 0.05).

Having determined the specificity of CCP-PIC-AF647 and its ability to detect small subpopulations of B cells, we used the combination of PICs to identify and characterize the primary ACPA-expressing B cells in the peripheral blood of five RA patients. We observed similar percentages of ACPA-expressing B cells in samples from individual donors stained with either SA-tetramers or PICs. The median frequency was determined at approximately 0.007% cells out of total B cells (Figure 5A,B). To assess whether the characteristics of the patient’s ACPA-expressing B cells were consistent with previous data, we also examined various B-cell subset-defining markers such as CD20, CD27, IgG-class B-cell receptors, and the activation surface marker CD80 (Figure 5B). A median of 50% of ACPA-expressing B cells detected by the PICs and SA tetramer was found to be positive for markers of MBCs, CD20 and CD27. Again, we found that both PICs and SA-tetramer stained ACPA-expressing B cells equally well. These findings show that PICs can serve as an alternative for SA tetramers for identifying and characterizing the ACPA-expressing B cells in RA patients.

### CCP and CD22 Ligand Co-functionalized PICs can Selectively Inhibit ACPA-Expressing B Cells

Having established the potential of PICs to detect ACPA-expressing B cells in RA patient samples, we set out to further exploit the modular character of PICs. In contrast to SA, PICs can accommodate multiple ligands on the same polymer backbone. We hypothesized that our antigen-carrying PICs could serve as an antigen-specific inhibitory moiety when conjugated to ligands that interact with inhibitory cell surface proteins. CD22, a B-cell-specific inhibitory receptor belonging to the SIGLEC family of lectins, is activated by recognition of α 2–6-linked sialic acids and regulates the Ca^2+^ signaling through phosphatases.^20^ By co-localizing ACPA BCRs with activated CD22 using liposomes containing CCP and a CD22 ligand (CD22L), Bednar et al. previously showed that ACPA secretion by B cells from RA patients was prevented.^21^ Additionally, the elegant use of polymeric multivalent antigens combined with CD22L for immunomodulatory applications has been shown in the literature.^22^ To study the ability of PICs to target inhibitory functions to autoreactive, ACPA-expressing B cells, we made use of Ramos B cells transfected with an ACPA BCR, as previously described.^23^ Importantly, these cells, unlike the immortalized B cells obtained from patients, only express membrane-bound BCRs and do not secrete IgG. Therefore, antibody–PIC immune complexes cannot form in solution, which could interfere with BCR activation through binding to the potent inhibitory receptor CD32/FcγRII. Hence, this cellular system allows the direct evaluation of CD22 targeting.

**Figure 6:**
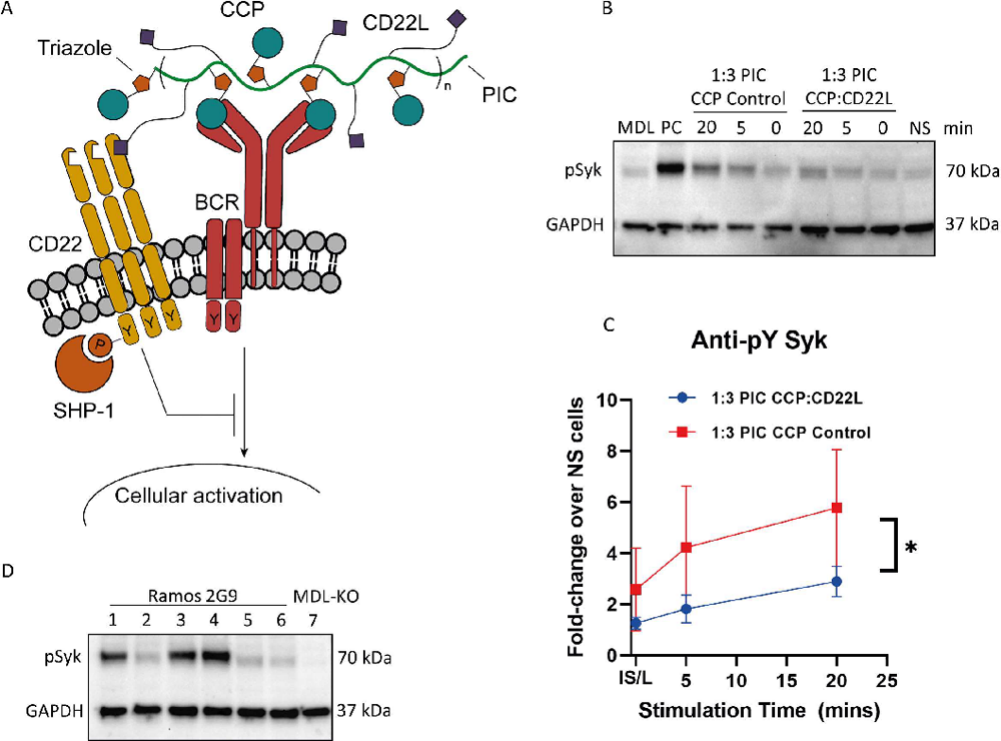
(A) PICs carrying both CCP and CD22L (A Neu5Ac α2-6, Gal β 1–4 Glc glycan moiety) can co-ligate the BCR and CD22 simultaneously and inhibit B-cell receptor activation. (B) Phospho-Syk expression in ACPA-expressing Ramos B cells stimulated with 80 nM of 1:3 CCP/CD22L PIC and 1:3 CCP control PIC for 5 and 20 min. IS/L (“immediate spin-down and lysis”) refers to cells that were treated with respective PICs, which immediately spun down and lysed. NS cells and MDL-KO (ACPA-B-cell receptor negative cells) were used as negative controls. Positive control consisted of ACPA-expressing Ramos B cells stimulated with 80 nM of 100% CCP PICs. (C) Average phospho-Syk expression in ACPA-expressing Ramos B cells stimulated with 80 nM of 1:3 CCP/CD22L PIC and 1:3 CCP control PIC and immediately spun down or stimulated for 5 and 20 min, determined from western blot quantification in three separate experiments expressed as fold-change over the phospho-Syk signal in NS ACPA-expressing Ramos B cells. Statistical differences between 1:3 CCP/CD22L PIC and 1:3 CCP control PIC were determined using a paired *t* test (*p* = 0.04). (D) Phospho-Syk expression in ACPA-expressing Ramos B cells stimulated with (1) 100% CCP 20 nM + 100% CD22L (60 nM), (2) 1:3 CCP/CD22L (80 nM), (3) 100% CCP (20 nM), (4) 100% CCP (80 nM), (5) 100% CD22L (80 nM), (6) no stimulus, and (7) MDL-KO (ACPA BCR negative) stimulated with 100% CCP (80 nM).

We detected high levels of CD22 expression on the surface of immortalized ACPA-expressing Ramos B cells (Figure S4). Next, we synthesized a DBCO-modified trisaccharide moiety (Neu5Ac α2-6, Gal β 1–4 Glc) that functions as a CD22 ligand^22^ (CD22L). PICs were functionalized to carry a ratio of 1:3 CCP to CD22L (1:3 CCP/CD22L PIC) (Figure 6A). To investigate the inhibitory effects of CD22L, CCP control PICs were also synthesized. These CCP control PICs carried the same amount of the antigen but lacked CD22L (1:3 CCP1 control PIC). To maximize the total peptide and ligand capacity on the PICs, CD22L-carrying PICs were functionalized with peptide-ligand:fluorophore ratio of 10:1. All PICs were synthesized using strain-promoted alkyne–azide cycloaddition click chemistry, as described before.^11,12^ To assess the levels of B-cell activation, we assessed tyrosine phosphorylation of Syk, a 72 kDa protein-tyrosine kinase expressed in B cells. Upon BCR engagement, a signaling cascade is initiated that results in the phosphorylation of Syk by autophosphorylation and phosphorylation by Lyn, an Src-family BCR-associated protein tyrosine kinase.^24^ In contrast, inhibitory signaling through CD22 can reduce the phosphorylation of Syk and thereby counter B-cell activation.

We stimulated ACPA-expressing Ramos B cells for 5 and 20 min with 1:3 CCP/CD22L and 1:3 CCP control PICs (Figure 6B) or spun them down and lysed immediately after adding the respective PICs (IS/L; immediate spin-down and lysis). Non stimulated cells (NS) served as a baseline for phospho-Syk expression. After 5 min of stimulation, we observed decreased phospho-Syk levels in cells treated with PICs that carried CD22L compared to cells treated with PICs lacking CD22L. The difference in phospho-Syk levels between the PICs was even more pronounced over time. Quantification of western blots obtained from three separate stimulation experiments confirmed the reduction in phospho-Syk expression mediated by the presence of PIC-conjugated CD22L (Figures 6C, S5).

Next, we questioned whether the co-localization of CCP and CD22L on the same PIC is necessary to elicit these inhibitory effects. Mechanistically, CD22 elicits its inhibitory function by physically associating with the BCR and recruiting downstream phosphatases.^25^ This indicates that co-ligation of the BCR and CD22 by the CCP/CD22L PICs could be limited by spatial restrictions.^22^ To test this hypothesis, we stimulated ACPA-expressing Ramos B cells for 5 min with 1:3 CCP/CD22L PICs and compared the phospho-Syk expression to cells stimulated with equimolar concentrations of 100% CCP and 100% CD22L on separate PICs and a PIC containing only CCP (Figure 6D). 1:3 CCP/CD22L PICs and the respective 100% CCP and CD22L PICs are not antigenically isodense. The level of phospho-Syk expression observed in response to stimulation with 20 nM of 100% CCP PIC was similar to the level observed after stimulating with 20 nM of 100% CCP PICs and 60 nM of 100% CD22L, indicating that the addition of CD22L PICs in itself does not reduce phospho-Syk expression. Moreover, cells stimulated with 80 nM of 1:3 CCP/CD22L PICs showed markedly lower phospho-Syk expression than the aforementioned PICs. These results not only confirm the experiments presented above but also suggest that co-localization of antigen and ligand is indeed required for antigen-specific B cell inhibition. Additionally, we observed that stimulating cells with 100% CD22L PICs does not induce phospho-Syk expression by itself, ruling out the possibility that these PICs interact with the BCR in a non-antigen specific manner. This observation corresponds to flow cytometry data, showing that the 100% CD22L PICs are not able to readily bind ACPA-expressing Ramos B cells in the absence of BCR stimulation (Figure S6). This lack of binding is likely explained by the fact that in resting B cells, CD22 is “masked” by high affinity cis–glycan interactions, mediated by CD22–CD22 homomultimeric complexes.^26^ These interactions not only “mask” CD22 from trans-interactions but also sequester CD22 away from the B-cell receptor.^25,27^ Upon B-cell receptor engagement, these interactions are disrupted and allow CD22 to be engaged by trans-ligands and thus can potentiate CD22’s inhibitory functions. All in all, these results suggest that PICs are suitable to not only phenotype rare B-cell populations but that they can also be applied in conjugation with ligands to elicit immunomodulatory effects.

## Conclusions

Here, we employed PICs for immunophenotypic and immunomodulatory purposes. PICs are novel synthetic polymers that, in solution, yield a semiflexible structure. The combination of semiflexibility and the length of PICs confers PICs with several advantages over conventional, non-semiflexible polymers, such as the prevention of forming random coils, allowing for more efficient multivalent binding and receptor clustering.^11,12^ We showed the utility of PICs carrying CCP in the detection of ACPA-expressing immortalized B-cell lines. Using an established double-staining flow cytometry approach,^15,17,18^ we were able to detect similar numbers and phenotypes of ACPA-expressing B cells with PICs and SA. The numbers of ACPA-expressing B cells detected with both reagents were in line with previous findings.^18^ The increased valency of CCP-PICs compared to the “gold-standard” CCP-SA did not yield an improved detection of ACPA-expressing B cells or of individual B-cell subsets, whereas lower background staining was observed. Based on what we observe in this manuscript and on unpublished observations, the effect of antigen valency on B-cell receptor binding likely follows a curve of diminishing returns. Nonetheless, the limited effect of increased valency is largely outweighed by their comparable specificity and remarkable properties of carriers of therapeutic compounds.

To demonstrate this, we here show that PICs can be used to inhibit B cells in an antigen-specific manner. PICs functionalized with 25% CCP and 75% of a trisaccharide CD22 ligand (CD22L) successfully inhibited BCR downstream signaling effects, as evidenced by reduced phospho-Syk expression. We also showed that co-localization of the CCP molecules and the CD22L molecules on the same PIC is required for the inhibitory effects observed. This underlines the importance of simultaneously and proximally co-ligating the BCR and CD22 to inhibit B cells. Although our results are promising in vitro, some hurdles have to be overcome before this modality can be used clinically. Mainly, the presence of autoantibodies in circulation might neutralize autoantigen-carrying PICs and reduce therapeutic efficacy. Combining PICs with antigen-shielding protection groups that can be locally unlocked by enzymes could solve this issue.^28^ Further studies are needed to investigate the PIC inhibitory effects, activation and inhibitory kinetics, and antigen-shielding strategies. Together, our data illustrate the application of PIC conjugates in the immunophenotyping and immunomodulation of rare B-cell populations.

## Supporting Information Available

Chemical synthesis steps of the various PICs, streptavidin and ligand molecules, and all experiments using cell lines and PBMCs (PDF)
