# Supplementary material for: Multifunctional, Multivalent PIC Polymer Scaffolds for Targeting Antigen-Specific, Autoreactive B Cells
Source: ACS Biomater Sci Eng. 2022 Mar 8;8(4):1486–93. doi: 10.1021/acsbiomaterials.1c01395 (PMC9006213; doi:10.1021/acsbiomaterials.1c01395)
Supplement: Supplementary file 1 — ab1c01395_si_001.pdf [file ab1c01395_si_001.pdf]

SUPPORTING INFORMATION

Multifunctional, multivalent PIC polymer scaffolds for targeting antigen-specific, autoreactive B cells

Hendy Kristyanto,[a,e] *Miles D. Holborough-Kerkvliet,*[a,e] *Lianne Lelieveldt,*[b] *Yvonne Bartels,*[b] *Roel Hammink,*[c, d] *Karin A.J. van Schie,*[a] *Rene E.M. Toes,*[a] *Kimberly M. Bonger,*[b*] *Hans Ulrich Scherer* [a*]

Address:

a) Department of Rheumatology, Leiden University Medical Center, Albinusdreef 2, 2333 ZA, Leiden, The Netherlands

b) Department of Synthetic Organic Chemistry, Radboud University, Heyendaalseweg 135, 6525 AJ Nijmegen, The Netherlands.

c) Department of Tumor Immunology, Radboud Institute for Molecular Life Sciences, Radboud University Medical Center, 6525 GA Nijmegen, Netherlands; Division of Immunotherapy,

d) Oncode Institute, Radboud University Medical Center, 6525 GA Nijmegen, Netherlands

e) Authors contributed equally

* Corresponding author: k.bonger@science.ru.nl and h.u.scherer@lumc.nl

MATERIALS AND METHODS

General synthetic methods and materials

Unless stated otherwise, all chemicals were used without further purification. If no further details are given the reaction was performed under ambient atmosphere and temperature. Low-resolution mass spectra (LRMS) were recorded on Thermo LCQ Advantage Max (ESI). A Thermo Finnigan LCQ Fleet ESI ion-trap mass spectrometer, which is equipped with a Shimadzu HPLC (C18-column, particle size 3 µm, acetonitrile/water gradient 5 - 100%, in 16 minutes and a flow of 0.2 ml/min) and a PDA detector, was used to separate organic compounds and record low-resolution mass spectra. High-resolution mass spectra (HRMS) of small molecules were recorded on a JEOL AccuTOF JMS-T100CS (ESI). Preparative HPLC was performed on a Shimadzu LC-20A Prominence system (Shimadzu, ’s-Hertogenbosch, The Netherlands) equipped with a Gemini NX-C18 column, 150 × 21.2 mm, particle size 10 µm (Phenomenex, Utrecht, The Netherlands). Gradient used was acetonitrile/water 5-50%, in 30 minutes and a flow of 6 ml/min. Analytical HPLC measurements were performed on a Shimadzu LC-20A Prominence system (Shimadzu, ‘s-Hertogenbosch, The Netherlands) equipped with a Gemini NX-C18 column, 150 × 3 mm, particle size 3 µm (Phenomenex, Utrecht, The Netherlands). Gradient used is acetonitrile/water 5 - 100%, in 30 minutes and a flow of 0.4 ml/min. Injected peptides were monitored at 254 nm and 215 nm and the desired peaks were integrated manually using a LabSolutions software package (Shimadzu, ‘sHertogenbosch, The Netherlands).

General Synthetic procedure of CCP peptides

CCP-Lys (**1**) and CArgP-Lys (**2**) were synthesized, cyclized and purified according to general procedures as previously described [1]. In short, the first amino acid, Fmoc-Lys(Mtt)-OH, (2 equiv.)

was added to the Wang resin with DIPCDI (2 equiv.), HOBt (4 equiv.) and DMAP (2 equiv.) in DMF. The mixture was shaken for 16h at room temperature. Upon completion, the resin was flushed three times with DMF and piperidine was then added for 30 min to cleave off the Fmoc protecting group. The resin was subsequently flushed three times with DMF. A mixture of 3 equiv. Fmoc-AA-OH, 3.6 equiv. HOBt and 3.3 equiv. DIPCDI was added to the resin to bind the subsequent amino acid. This reaction was incubated for 30 min at room temperature. After coupling of the next amino acid, the remaining free amines were capped with acetic anhydride (1 ml) and pyridine (1 ml) in DMF (12 ml). After washing three times with DMF, piperidine was added again and the cycles continued. After the last amino acid, chloroacetic anhydride (5 equiv.) and DIPEA (5 equiv.) were added in DMF and shaken for 45 min. Finally, a mixture of 92.5% TFA, 2.5% H2O, 2.5% EDT and 2.5% TIPS was made. This mixture was added to the resin and incubated for 3h at room temperature to cleave off the peptide from the resin and to deprotect the amino acid residues. The peptide was precipitated in diethyl ether, filtered and dried. Kaiser tests were performed to follow the coupling reactions.

General procedure for peptide cyclisation

The crude peptides were dissolved in a 50 mM NH_4HCO_3 buffer pH 8.4: MeCN 1:1, at a concentration of 2 mg/ml and stirred for 24h. MeCN was evaporated and the remaining H_2O was lyophilized. The peptides were purified using preparative reversed-phase HPLC and analyzed using analytical HPLC.

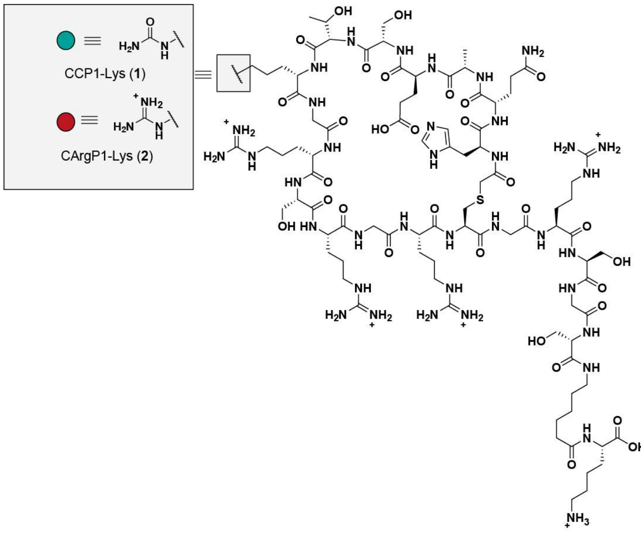

CCP-Lys (1). HPLC: Rt. 10.209 min. LCMS (ESI+) m/z calcd for $C_{90}H_{157}N_{39}O_{32}S^{2+}$ $[M+2H]^{2+}$ 1164.08, found 1163.00. $C_{90}H_{158}N_{39}O_{32}S^{3+}$ $[M+3H]^{3+}$ 776.39, found 776.92. $C_{90}H_{159}N_{39}O_{32}S^{4+}$ $[M+4H]^{4+}$ 582.54, found 583.00.

CArgP-Lys (2). HPLC: Rt. 10.310 min. LCMS (ESI+) m/z calcd for $C_{90}H_{159}N_{40}O_{31}S^{3+}$ $[M+3H]^{3+}$ 776.06, found 776.64. $C_{90}H_{160}N_{40}O_{31}S^{4+}$ $[M+4H]^{4+}$ 582.30, found 582.68.

DBCO functionalization. All peptides with a C-terminal lysine were functionalized with a NHS-PEG$_4$-DBCO after cleavage and cyclization. The lysine-containing peptides **1** and **2** (2.0 µmol) were dissolved in DMF (2.0 mL) and DIPEA (204 µmol, 35.5 µL, 100 equiv.) was added. NHS-PEG$_4$-DBCO (16.2 mg, 25µmol) was dissolved in DMSO (100 µL, 250 mM) and 20 µL of the stock solution was added (resulting in a final concentration of 2.5 mM, 2.5 equiv.). The reaction was stirred for 5 hours at r.t. before the product was precipitated in Et_2O. The pellet was washed three times and dried afterwards.

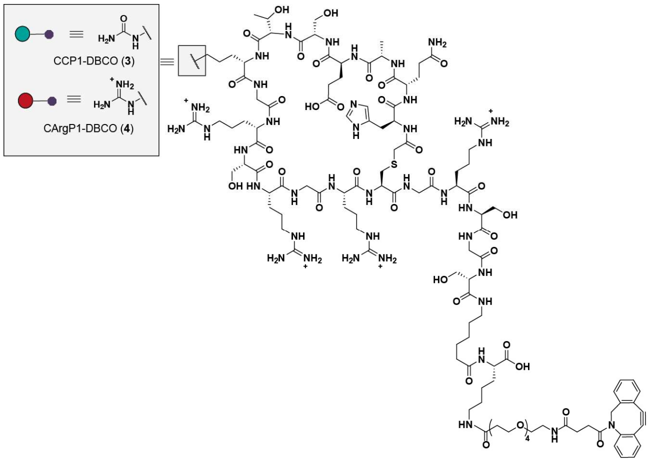

CCP1-PEG4-DBCO (3). CCP-DBCO was synthesized following the procedures described in the general DBCO-peptide functionalization. CCP-Lys (**1**) was used as starting material. HPLC: Rt. 16.551 min. LCMS (ESI+) m/z calcd for $C_{120}H_{191}N_{41}O_{39}S^{2+}$ $[M+2H]^{2+}$ 1431.20, found 1432.08. $C_{120}H_{192}N_{41}O_{39}S^{3+}$ $[M+3H]^{3+}$ 954.47, found 955.36. $C_{120}H_{193}N_{41}O_{39}S^{4+}$ $[M+4H]^{4+}$ 716.10, found 718.36.

CArgP1-PEG4-DBCO (4). CArgP-DBCO was synthesized following the procedures described in the general DBCO-peptide functionalization. CArgP-Lys (**2**) was used as starting material. HPLC: Rt. 16.367 min. LCMS (ESI+) m/z calcd for $C_{120}H_{192}N_{42}O_{38}S^{2+}$ $[M+2H]^{2+}$ 1430.70, found 1431.52. $C_{120}H_{193}N_{42}O_{38}S^{3+}$ $[M+3H]^{3+}$ 954.14, found 955.08. $C_{120}H_{194}N_{42}O_{38}S^{4+}$ $[M+4H]^{4+}$ 715.86, found 717.48.

Biotin functionalization

Biotinylated cyclic citrullinated peptide (CCP-biotin, **5**) and its arginine control variant (CArgP-biotin, **6**) were synthesized, cyclized and purified according to procedures as previously described.[1] In short, the first amino acid, Fmoc-Lys(Mtt)-OH, (2 equiv.) was added to the Wang resin with DIPCDI (2 equiv.), HOBt (4 equiv.) and DMAP (2 equiv.) in DMF. The mixture was shaken for 16h at room temperature. After washing, the Mtt group was cleaved off using 2% TFA in DCM repeatable for 2 minutes. After washing with DCM and DMF, Biotin was coupled using

DIPCDI (3.3 equiv.) and HOBt (3.6 equiv.). The peptides were further synthesized and cyclized according to the general procedure as described above.

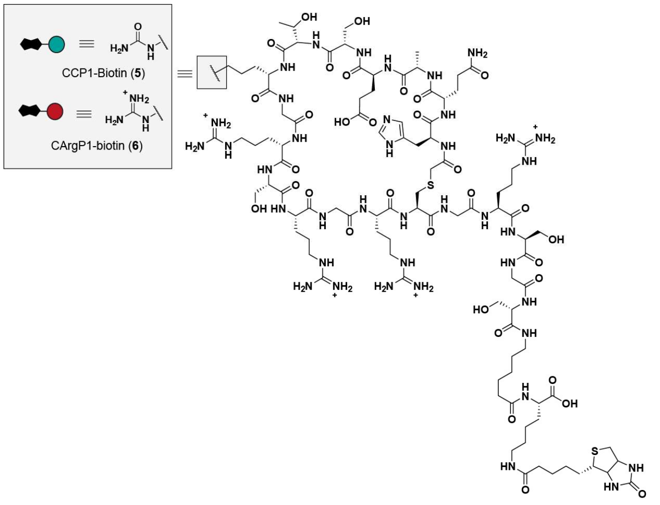

CCP-biotin (5). HPLC: Rt. 12.753 min. LCMS (ESI+) m/z calcd for $C_{100}H_{171}N_{41}O_{34}S_2^{2+}$ $[M+2H]^{2+}$ 1277.61, found 1278.08. $C_{100}H_{172}N_{41}O_{34}S_2^{3+}$ $[M+3H]^{3+}$ 852.07, found 852.68. $C_{100}H_{173}N_{41}O_{34}S_2^{4+}$ $[M+4H]^{4+}$ 639.31, found 641.16.

CArgP-biotin (6). HPLC: Rt. 12.731 min. LCMS (ESI+) m/z calcd for $C_{100}H_{172}N_{42}O_{33}S_2^{2+}$ $[M+2H]^{2+}$ 1277.13, found 1277.56. $C_{100}H_{172}N_{41}O_{34}S_2^{3+}$ $[M+3H]^{3+}$ 851.75, found 852.28. $C_{100}H_{173}N_{41}O_{34}S_2^{4+}$ $[M+4H]^{4+}$ 639.06, found 640.20.

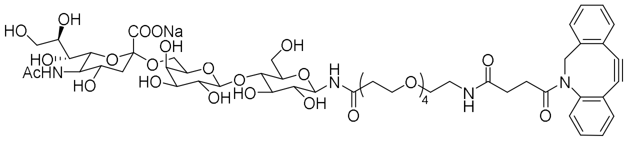

CD22L-PEG$_4$-DBCO (7). The CD22L-PEG$_4$-DBCO was synthesized by partly adapting a previous published protocol [2]. Briefly, 6'sialyllactose sodium salt (44 mg, 0.067 mmol) was dissolved in 2.5 mL MilliQ and heated to 37 ºC, in an open tube. Next, NH_4HCO_3 was added portion wise over the course of 3 days (10x added, 300 mg, 3 mmol per addition), after which 25 ml MilliQ was added. The solvent was evaporated and again 25 ml MilliQ was added, followed

by evaporation of the solvent, resulting in an off white solid (31.7 mg, 0.048 mmol, 72%). The crude amine product was dissolved in 1 mL borate buffer (pH 8.4, 50 mM), and 0.75 eq of DBCO-PEG_4-NHS (23.6, 0.036 mmol, 100 mM stock in DMSO) was added. The reaction was mixed at room temperature for 1 day, after which the solution was precipitated in acetonitrile. The product was purified using HPLC, with a triethylammonium bicarbonate buffer/ methanol solvent system, (2.76 mg, 0.0023 mmol, 6.3 %) Maldi-ToF m/z calcd for $C_{53}H_{73}N_4NaO_{25}$ $[M+H]^+$ 1189.454 found 1189.851

Modification of PICs

PICs scaffold were synthesized according to previously published methods [3, 4]. The PICs were kept on ice at all times and stored in non-stick microfuge tubes. DIBO-AF647, DIBO-AF594 and DIBO-AF405 were dissolved in DMSO (1 mM). 4.90 µl of DIBO-AF647 stock solution was added to PICs (1.325 ml, 1 mg/ml). The reaction was kept at 4 °C overnight in a shaker. Afterwards, 50 µl was used for subsequent modification with a single antigen peptide. To this 50 µl, 8.50 µl of antigen peptide (2 mg/ml in PBS, 1.5 equiv. compared to available azides) was added. Next, PBS (8.50 µl) was added to have a final PIC concentration of 0.85 mg/ml. The reaction was again left overnight at 4°C.

Before using these scaffold in flow cytometry measurements, stock solutions of around 1 µM peptide concentration were prepared. Peptide concentrations were calculated starting with the initial concentration (mg/ml) and the molecular weight of a monomer (360 Da, weight of monomers stays the same after polymerization). From this 2.36 mM of monomer ($\frac{0.85\ mg/mL}{360} \times 1000$), one in every 83 monomers contains an azide. Maximal peptide concentration on the PICs is therefore 28.44 µM ($\frac{2.36\ mM}{83} \times 1000$). This mixture was then diluted to obtain the desired starting stock concentration for serial dilutions

Functionalization of PIC with CD22 ligand

To 50 µl of PIC (1 mg/ml in PBS) were added different ratios of CCP-DBCO (**3**) and CD22L. The total reactant was kept constant at 1 eq compared to available azides on the PIC scaffold. For 100% CCP1 2.8 µl of CCP1 (2 mg/ml in PBS) was added, for 75% CCP1/25% CD22L 2.1 µl of CCP1 and 0.55 µl CD22L (1.08 mg/ml in MilliQ), for 50% CCP1/50% CD22L 1.4 µl of CCP1 and 1.1 µl CD22L, for 25% CCP1/75% CD22L 0.7 µl of CCP1 and 1.65 µl CD22L and for 100% CD22L 2.2 µl CD22L. The mixtures were stirred overnight at 4 ºC and used without further purification.

Peptide loading onto streptavidin

Streptavidin (SA) fluorophore conjugates were purchased from Thermo Fisher. The proteins were dissolved in PBS (2 mg/ml) and 116 µl biotinylated peptides **5** or **6** (5 mg/ml in PBS, 50 equiv.) were added to 125 µl of SA. The reaction was left overnight at 4 °C and the mixtures were purified afterwards using 40K Zeba spin desalting column to remove the excess of biotinylated peptide added. Protein concentrations were determined afterwards using UV-Vis measurements. Absorbance was measured at 280 nm and 654 nm. Using Lambert-Beers law (Abs = c * ε * d), fluorophore concentration was calculated, ε for AF647 is 270,000 M^{-1} cm^{-1}, d is 0.1 cm. For SA the absorbance was first corrected for the fluorophore attached. The calculations were made using: $Abs_{SA} = Abs_{280} - Cf * Abs_{fluorophore}$ (in which Cf is the correction factor for AF647 and is 0.03). Using this corrected absorbance, the concentration was calculated according to the law of Lambert-Beer, using ε for SA is 167,000 M^{-1} cm^{-1}. The correlation between the corrected SA concentration and the concentration at the wavelength of the fluorophore depicts the ratio of SA:fluorophore.

Patients and healthy donors

Peripheral blood of ACPA-positive RA patients was obtained at the outpatient clinic of the Department of Rheumatology at Leiden University Medical Centre (LUMC). All RA patients fulfilled the 2010 ACR/EULAR criteria for RA at the time of diagnosis. Treatment regimens included conventional disease-modifying anti-rheumatic drugs and glucocorticoids. Both patients and healthy donors gave written informed consent.

Cell isolation and culture

To determine which ratio of CCP and fluorophore in PIC to use in flow cytometry experiments, ACPA-expressing HEK 293T cells ($HEK^{ACPA\text{-}TM}$) were generated by transducing HEK 293T cells with ACPA-encoding lentiviral vectors, as previously described [5]. These cells were cultured in Dulbecco Modified Eagle Medium (DMEM) supplemented with 8% heat-inactivated fetal calf serum (FCS) , penicillin/streptomycin (PS, 100 U/ml), 2 mM Glutamax (Glut) and 1 µg/ml puromycin (InvivoGen).

To assess for the staining specificity of both fluorescently labelled CCP-PIC scaffolds and SA (streptavidin), human immortalized ACPA and tetanus toxoid (TT)-expressing AIMM B cells were used. These cell were immortalized by transducing antigen-specific B cells with Bcl-6 and Bcl-XL-encoding retroviruses, as previously described [6, 7].

To assess the capacity of fluorescently labeled CCP1-PIC and SA in detecting rare ACPA-expressing B cells, 10,000 immortalized ACPA-expressing B cells were mixed with 10 million peripheral blood mononuclear cells (PBMC) from healthy donors. PBMC were isolated from 50 ml of heparinized peripheral blood using Ficoll-Paque gradient centrifugation and stored overnight at 4 °C in Iscove's Modified Dulbecco's Medium (IMDM) supplemented with 8% FCS, 100 U/ml PS and 2 mM Glutamax.

To identify and characterize ACPA-expressing B cells in RA blood, PBMC were isolated from peripheral blood of RA donors as above.

For stimulation experiments, ACPA-expressing Ramos B cells were used. Ramos cells were transduced with ACPA-encoding lentiviral vectors[8]. Ramos cells were cultured in RPMI 1640 (Thermofisher) supplemented with 8% FCS, 100 U/ml PS, 2 mM GlutaMax and 10 mM HEPES).

Flow cytometry

To determine which ratio of CCP and fluorophore in PIC to use, two ratios of CCP-AF647 were tested, i.e. 10:1 and 2:1. 100,000 $HEK^{ACPA-TM}$ and HEK^{WT} cells were stained with 7 concentrations of AF647 labelled CCP-PIC for 30 minutes on ice, washed and fixed with 1% paraformaldehyde before measurement. To compare, AF647-labelled CCP-SA at the similar concentrations was used.

To assess staining specificity, 100,000 immortalized ACPA-expressing and TT-specific B cells were stained with either a combination of 85 nM of AF647-labeled CCP-PIC, AF594-labeled CCP-PIC and AF405-labeled CArgP-PIC or a combination of 100 nM of AF647-labeled CCP-SA, AF594-labeled CCP-SA and AF405-labeled CArgP-SA for 30 minutes on ice, washed and fixed with 1% paraformaldehyde before measurement. The cells were considered ACPA-expressing if they were double positive for two differently labelled CCP-PIC or CCP-SA and negative for AF405-labeled CArgP-PIC or -SA.

To assess the capacity of fluorescently labeled CCP-PIC and SA in detecting rare ACPA-expressing B cells, 10,000 immortalized ACPA-expressing B cells were mixed with 10 million PBMC from healthy donors, stained for the presence of dead cells using fixable violet dead cell stain kit (Molecular Probes) for 30 minutes on ice, washed and divided into two fractions. The first fraction was treated with 10 μM of unlabeled CCP-SA and the second with 1% bovine serum albumin (BSA, from Sigma)-containing phosphate buffered saline (PBS, pH 7.4) on ice. After 30

minutes, without washing, each fraction was subdivided into two, one was stained with a combination of (final concentration) 85 nM of AF647-CCP-PIC, AF594-CCP-PIC and AF405-CArgP-PIC and the other with a combination of (final concentration) 100 nM of AF647-CCP-SA, AF594-CCP-SA and AF405-CArgP-SA. All fractions were also stained with anti-CD3 Pacific Blue (PB, clone UCHT1), anti-CD14 PB (clone M5E2), anti-CD19 APC-Cy7 (clone Sj25C1, all from BD Biosciences) for 30 minutes on ice, washed and fixed with 1% paraformaldehyde.

To compare the capacity of labelled CCP-PIC and CCP-SA to identify ACPA-expressing B cells in RA blood, PBMC from RA patients were stained for the presence of dead cells as above and divided into two fractions. One was sequentially stained with the combination of labelled CCP/CArgP-PIC, the other with SA as above. Both fractions were also stained with anti-CD3, anti-CD14, anti-CD19 as above and anti-CD20 AF700 (clone 2H7, Biolegend), anti-CD27 PE-Cy7 (clone M-T271), anti-CD80 Brilliant Blue 515 (clone L307.4), anti-IgG Brilliant Violet 510 (BV510, clone G18-145, all except CD20 from BD Biosciences) for 30 minutes on ice, washed and fixed with 1% paraformaldehyde.

To assess the expression of CD22 on immortalized ACPA-expressing B cell lines (Ramos), 100,000 cells were stained for dead cells as above, washed, stained with either anti-CD22 (clone HIB22, BD bioscience) or the isotype antibody and fixed with 1% paraformaldehyde.

Most flow cytometry measurements were conducted using a BD LSR Fortessa (BD Biosciences). Data analyses were conducted using BD FACSDIVA software (version 8.0.2) and FlowJo software (version 10.2).

B cell stimulation assays

To assess the capacity of CCP, CD22L conjugated PICs to inhibit B cell activation, we stimulated 500,000 cells with 80 nM of PICs before immediately spinning down and lysing (IS/L), or stimulated cells for 5 or 20 minutes. Cells were stimulated at 37 °C and were spun down at 13,000 RPM at 4 °C after the respective stimulation times. Supernatant was taken off and lysis buffer consisting of NP-40 and Protease/Phosphatase Inhibitor Cocktail (100X) (Cell Signaling) was applied to the cells. Cells were lysed for 30 minutes at 4 °C and subsequently spun down for 20 minutes at 13,000 RPM and 4 °C before transferring to new Eppendorf tubes.

Western Blot

Lysates from the B cell stimulation assays were mixed in with Laemmli buffer (4x) and heated up for 10 minutes at 95 °C. Samples were run in Bio-Rad precast gels (4-15%) in a TGS running buffer for 1.5 hours at 100 V. Subsequently, the gels were rinsed in milliQ for 5 minutes and transferred to Transblot Turbo Mini 0.2 µm nitrocellulose membranes using a Bio-Rad Transblot Turbo system. Blots were washed 3 times for 5 minutes in PBT (PBS, 0.05% Tween) and blocked in PTE (PBS, 0.05% Tween & 3% Skim milk powder) for 1 hour. Blots were washed 3 times for 5 minutes in PBT and incubated with p-Syk antibody (#2717S, Cell Signaling) and GAPDH antibody (#MAD374, Sigma-Aldrich) overnight at 4 °C in PTE. Blots were rinsed twice in PBT before incubating in polyclonal secondary anti-mouse and anti-rabbit antibodies from Agilent/Dako for 1 hour at room temperature. Blots were washed 3 times for 5 minutes prior to adding ECL (Pierce ECL Blotting Substrate, Thermo Scientific) and visualized on a Bio-Rad ChemiDoc Imager.

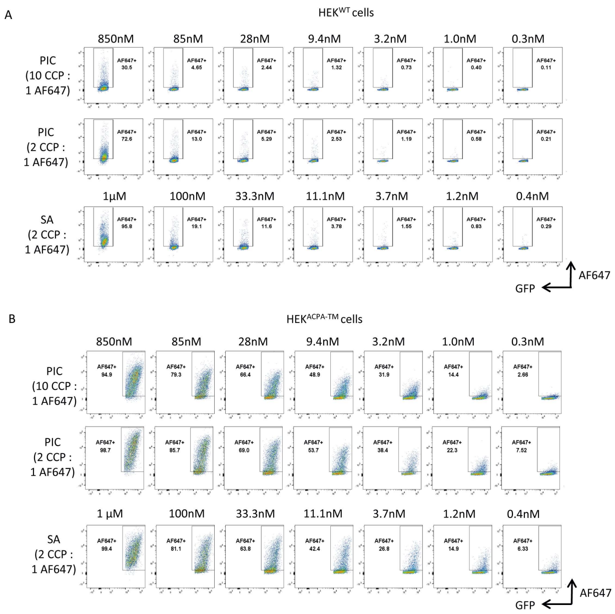

Figure S1. Specificity of ACPA-expressing HEK cell detection using CCP-PIC-AF647 and CCP-SA-AF647. Flow cytometry binding titration of PICs with different CCP and AF647 ratios, and SA showing percentage of AF647-positively stained cells on A) wild-type HEK (HEK^{WT}) cells and B) ACPA-expressing HEK ($HEK^{ACPA\text{-}TM}$) cells

A

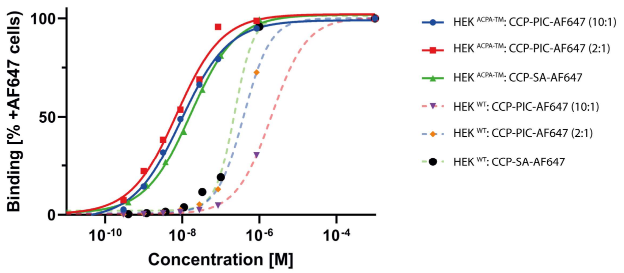

B

	CCP-PIC-AF647 (10:1)	CCP-PIC-AF647 (2:1)	CCP-SA-AF647
Kd (HEK $^{ACPA\text{-}TM}$) (nM)	9.6	7.3	16.5
Kd ($^{HEK\ WT}$) (nM)	2064	394	228
Fold-change HEK $^{ACPA\text{-}TM}$/HEK ^{WT}	215.0	54.2	13.8
Fold-change PIC/SA	15.5	3.9	1

Figure S2. A) Concentration (M) of respective construct plotted on the X-axis versus percentage of AF647-positive wild-type HEK (HEK^{WT}) cells and ACPA-expressing HEK (HEK$^{ACPA\text{-}TM}$) on the Y-axis. Data points were based on FACS plots shown in Supplementary Figure 1. Fitting curves (A) and Kd values (B) were obtained from GraphPad Prism calculations (Non-linear regression, [Agonist] versus response, Four parameters). ‘Fold-change HEK$^{ACPA\text{-}TM}$ /HEK^{WT}’ refers to the ratio of the Kd calculated for the binding of a construct to HEK$^{ACPA\text{-}TM}$ divided by the EC50 of the same construct calculated for HEK^{WT}. ‘Fold-change PIC/SA’ is obtained by dividing the ‘Fold-change HEK$^{ACPA\text{-}TM}$/HEK^{WT}’ value for SA by the values for the respective CCP-PIC-AF647 constructs.

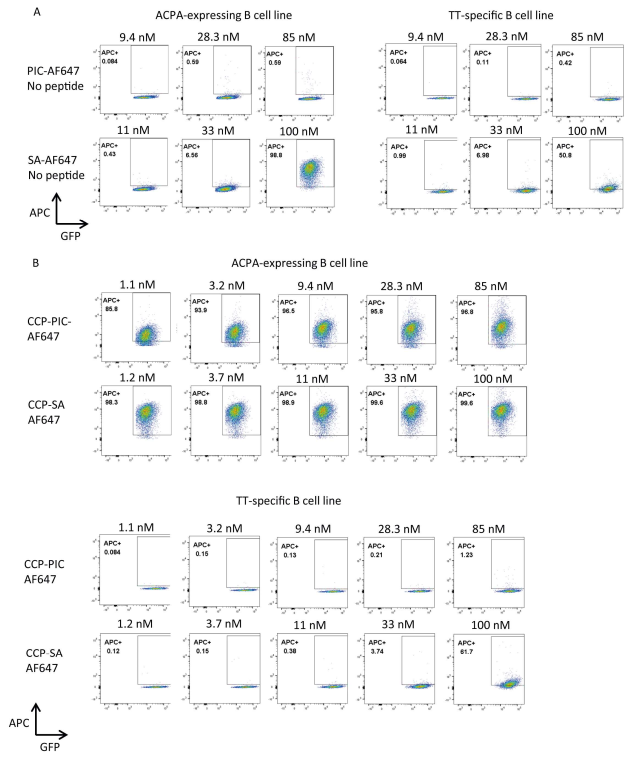

Figure S3. A) Specificity of ACPA-expressing B cell detection using fluorescently labelled CCP-PIC-AF647 and CCP-SA-AF647 on ACPA-expressing and TT-specific immortalized primary B cells. B) Specificity of ACPA-expressing B cell detection using fluorescently labelled CCP-PIC-AF647 and CCP-SA-AF647 on ACPA-expressing and TT-specific immortalized primary B cells.

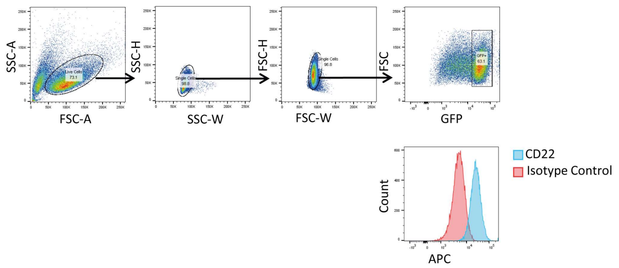

Figure S4. Ramos ACPA-expressing B cells express CD22. The gating strategy and expression of CD22 (blue) on Ramos ACPA-expressing B cells compared to an appropriate APC-labelled isotype control (red).

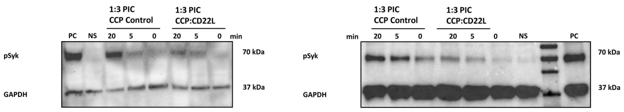

Figure S5. The two remaining western blots used to quantify the fold-changes of phospho-Syk over non-stimulated conditions in Figure 6C. Phospho-Syk expression in ACPA-expressing Ramos B cells stimulated with 80 nM of 1:3 CCP:CD22L PIC and 1:3 CCP Control PIC for 5 and 20 minutes. IS/L ("immediate spin-down and lysis") refers to cells that were treated with respective PICs and immediately spun down and lysed. Non stimulated (NS) cells and MDL-KO (ACPA-B cell receptor negative cells) were used as negative controls. PC (positive control) consisted of ACPA-expressing Ramos B cells stimulated with 80 nM of 100% CCP PICs.

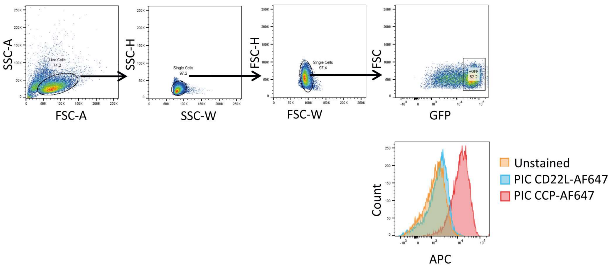

Figure S6. PICs conjugated to only CD22L (100% CD22L PIC) do not bind to ACPA-expressing Ramos B cells (blue), compared to unstained cells (orange, negative control) and 100% CCP PIC (red, positive control).

REFERENCES

1. Lelieveldt, L.; Kristyanto, H.; Pruijn, G. J. M.; Scherer, H. U.; Toes, R. E. M.; Bonger, K. M., Sequential Prodrug Strategy To Target and Eliminate ACPA-Selective Autoreactive B Cells. *Mol Pharm* **2018,** *15* (12), 5565-5573.
2. Tsuchida, A.; Kobayashi, K.; Matsubara, N.; Muramatsu, T.; Suzuki, T.; Suzuki, Y., Simple synthesis of sialyllactose-carrying polystyrene and its binding with influenza virus. *Glycoconj J* **1998,** *15* (11), 1047-54.
3. Mandal, S.; Eksteen-Akeroyd, Z. H.; Jacobs, M. J.; Hammink, R.; Koepf, M.; Lambeck, A. J. A.; van Hest, J. C. M.; Wilson, C. J.; Blank, K.; Figdor, C. G.; Rowan, A. E., Therapeutic nanoworms: towards novel synthetic dendritic cells for immunotherapy. *Chem Sci* **2013,** *4* (11), 4168-4174.
4. Mandal, S.; Hammink, R.; Tel, J.; Eksteen-Akeroyd, Z. H.; Rowan, A. E.; Blank, K.; Figdor, C. G., Polymer-based synthetic dendritic cells for tailoring robust and multifunctional T cell responses. *ACS chemical biology* **2015,** *10* (2), 485-92.
5. Kerkman, P. F.; Fabre, E.; van der Voort, E. I.; Zaldumbide, A.; Rombouts, Y.; Rispens, T.; Wolbink, G.; Hoeben, R. C.; Spits, H.; Baeten, D. L.; Huizinga, T. W.; Toes, R. E.; Scherer, H. U., Identification and characterisation of citrullinated antigen-specific B cells in peripheral blood of patients with rheumatoid arthritis. *Annals of the rheumatic diseases* **2015**.
6. Kwakkenbos, M. J.; Diehl, S. A.; Yasuda, E.; Bakker, A. Q.; van Geelen, C. M.; Lukens, M. V.; van Bleek, G. M.; Widjojoatmodjo, M. N.; Bogers, W. M.; Mei, H.; Radbruch, A.; Scheeren, F. A.; Spits, H.; Beaumont, T., Generation of stable monoclonal antibody-producing B cell receptor-positive human memory B cells by genetic programming. *Nat Med* **2010,** *16* (1), 123-8.
7. Germar, K.; Fehres, C. M.; Scherer, H. U.; van Uden, N.; Pollastro, S.; Yeremenko, N.; Hansson, M.; Kerkman, P. F.; van der Voort, E. I. H.; Reed, E.; Maassen, H.; Kwakkenbos, M. J.; Bakker, A. Q.; Klareskog, L.; Malmstrom, V.; de Vries, N.; Toes, R. E. M.; Lundberg, K.; Spits, H.; Baeten, D. L., Generation and characterization of anti-citrullinated protein antibody-producing B-cell clones from rheumatoid arthritis patients. *Arthritis & rheumatology (Hoboken, N.J.)* **2018**.
8. Kissel, T.; Reijm, S.; Slot, L. M.; Cavallari, M.; Wortel, C. M.; Vergroesen, R. D.; Stoeken-Rijsbergen, G.; Kwekkeboom, J. C.; Kampstra, A.; Levarht, E.; Drijfhout, J. W.; Bang, H.; Bonger, K. M.; Janssen, G.; van Veelen, P. A.; Huizinga, T.; Scherer, H. U.; Reth, M.; Toes, R., Antibodies and B cells recognising citrullinated proteins display a broad cross-reactivity towards other post-translational modifications. *Ann Rheum Dis* **2020,** *79* (4), 472-480.
